# Impact and Post-Impact Performance of Sandwich Wall Boards with GFRP Face Sheets and a Web-Foam Core: The Effects of Impact Location

**DOI:** 10.3390/ma11091714

**Published:** 2018-09-13

**Authors:** Yiwei Xia, Xiaoping Li, Yu Peng, Mianheng Lai, Lu Wang

**Affiliations:** 1 College of Civil Engineering, Nanjing Tech University, Nanjing 211816, China; xiayiwei323@163.com (Y.X.); 15050530924@163.com (X.L.); 2 Nanjing Jianye District Government Office, Nanjing 210009, China; 13505145621@163.com; 3 School of Civil Engineering, Guangzhou University, Guangzhou 510006, China; laimianheng@gzhu.edu.cn

**Keywords:** impact testing, sandwich wall boards, impact location, compressive strength, post impact behavior

## Abstract

In recent years, load-bearing exterior sandwich wall boards have been adopted in civil engineering. The exterior walls of structures are often exposed to low velocity impacts such as stones, tools, and windborne debris, etc. The ultimate loading capacity, deformation, and ductility of sandwich walls are weakened by impact loads. In this study, the sandwich wall boards consisted of glass fiber reinforced plastic (GFRP) face sheets and a web-foam core. The core of wall boards was not the isotropic material. There was no doubt that the mechanical performance was seriously influenced by the impact locations. Therefore, it is necessary to carry out an investigation on the impact and post-impact performance of exterior wall boards. A comprehensive testing program was conducted to evaluate the effects of impact locations and impact energies on the maximum contact load, deflection, and contact time. Meanwhile, the compression after impact (CAI) performance of wall boards were also studied. The results indicated that the impact location significantly affects the performance of wall boards. Compared with an un-damaged wall board, the residual ultimate loading capacity of damaged wall boards reduced seriously, which were not larger than 50% of the designed ultimate loading capacity.

## 1. Introduction

As the load-bearing components, sandwich wall boards have been widely adopted in civil engineering because of their advantages of large strength to weight ratio, excellent anti-corrosion ability, and thermal insulation property. Sandwich wall panels, consisting of two thin, fiber-reinforced plastic face sheets and light-weight core material such as polystyrene foam, could be fabricated to obtain a high ultimate strength [1].

**Figure 1:**
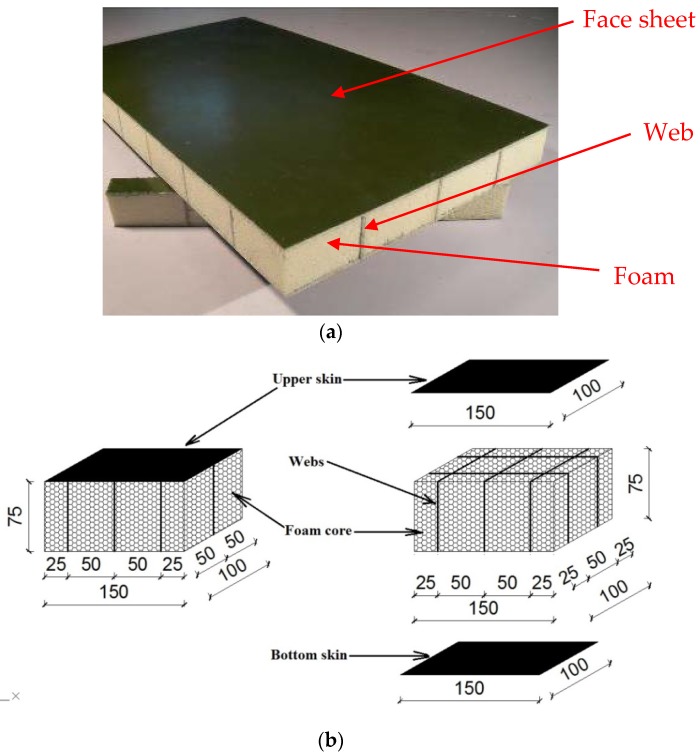
Composite sandwich wall board.

In the past 10 years, a lot of studies of the performance of sandwich wall boards have been carried out [2,3,4,5,6,7,8,9,10]. Shipsha and Zenkert [11] conducted an experimental study on compression after impact (CAI) strength of foam-cored sandwich panels. The impact damage was mainly foam core crushing accompanied by a permanent indentation in the face sheet. Meanwhile, residual dent growth was observed by digital speckle photography analysis. CAI strength of sandwich panels decreased with increasing impact damage size. Moreover, a finite element model was developed to predict the CAI strength. Zhao et al. [12] carried out an investigation to evaluate the influence of low velocity impact on honeycomb-cored sandwich structures. The test results indicated that the impact damage led to a change in failure modes; in the meantime, the compressive failure strength decreased by 10%. A finite element model was also established to evaluate the CAI behavior of specimens. The simulation results showed that the failure process of the un-damaged and damaged specimens under compressive loading was similar in experimental phenomena. Vaidya et al. [13] evaluated the low-velocity impact response of sandwich wall boards. The results showed that the rear face sheet of the wall board can be observed to be un-damaged when exposed to impact loading. Meanwhile, the proposed wall boards can prevent blunt object projectile penetration when the impact velocity is less than 135 m/s. Schubel et al. [14] investigated impact and post-impact performance of sandwich wall boards. The CAI tests were conducted compared with un-damaged specimens. The test results showed that the compressive strength of specimens decreased by 50% due to the delamination damage. Kenny and Torre [15] proposed a new corrugated sandwich wall panel. The test results showed that this panel can absorb impact energy around 35% more than that of traditional material, but a large number of tests have to be conducted to obtain reliable material properties before conducting the design of composite sandwich wall panels. In our companion paper [16,17,18], authors developed a new sandwich wall board made of glass fiber reinforced plastic (GFRP) face sheets and web-foam, as shown in Figure 1a. The interfacial delamination can be avoided by using GFRP webs. A large number of tests were conducted to evaluate the mechanical properties of this new wall board. The test results demonstrated that, compared to normal foam-cored sandwich wall boards, the ultimate axial strength increased roughly by 140%.

However, the core of developed wall boards in this study was not the isotropic material. Besides the fact that the ultimate strength of sandwich wall boards was significantly affected by impact loading, there was no doubt that the failure mode and post impact performance were seriously influenced by the impact locations. Li et al. [19] used composite sandwich wall boards strengthened by both T-shaped and I-shaped stiffeners to conduct on-edge impact testing. The test results indicated that the impact damage of wall boards impacted on the free edge was more serious than that of wall panels impacted on face sheets, because the edge-impacted region lacked surrounding constraints. Besides the studies conducted by Li, hardly any references have been collected that evaluate the effects of impact location on the behavior of sandwich wall boards. Therefore, it is necessary to evaluate the low-velocity impact response and the residual axial load capacity of composite sandwich wall boards with different impact locations. This is the reason why we conducted this investigation. In this study, a test program was carried out to study the effects of impact locations, impact energies, and foam densities on the maximum contact force, deflection, and contact time. In the meantime, the CAI tests were also conducted to study the residual strength of the composite sandwich wall boards.

## 2. Materials and Methods

### 2.1. Specimens

70 specimens, manufactured by a vacuum-assisted resin infusion process, were tested, with five replicates for each condition. The face sheets were made by the E-type glass fiber fabric with density of 800 g/m^2^ and the unsaturated polyester resin. The specimens were filled polyurethane foams with different densities (40, 60 and 100 kg/m^3^). The width (*w* = 100 mm), length (*d* = 200 mm), and face sheet thickness (*t* = 2.4 mm) of all wall boards were identical.

Specimens 0D1E0 and L0D1E0 were control specimens, which did not subject to the low-velocity impact. They were used to prove the axial compressive behavior of the “perfect” sandwich wall panels. Specimens 0D1E2, 0D1E5, 0D1E8, 0D4E8, and 0D6E8 were control wall boards without webs to show the low-velocity impact response of the foam-cored sandwich wall boards. The other wall boards were reinforced with webs with different impact location (L), foam density (D), and impact energy (E). Figure 1b shows the dimension of specimens with web-foam core. Table 1 summarizes the specimen details.

**Figure 2:**
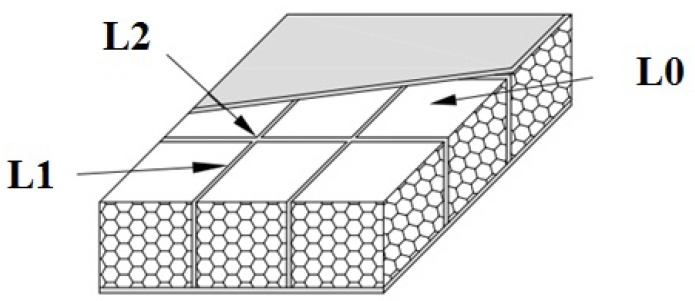
Impact locations.

1. Impact location with either skin, web, or cross of webs, designated as L0, L1, and L2, respectively, as shown in Figure 2.
2. Foam density with either 40, 60, and 100 kg/m^3^, designated as D4, D6, and D1, respectively.
3. Impact energy with either 27, 54, or 81 J, designated as E2, E5, and E8, respectively.

### 2.2. Material Properties

Tensile and compressive properties of GFRP face sheets were tested according to ASTM D3039/D3039M-08 [20] and ASTM D695-10 [21], respectively. The thickness of coupons was equal to the thickness of face sheet. During the tests, the displacement control was adopted. The displacement rate was 2 mm per min. The material properties of face sheet are summarized in Table 2. Six foam cubics with the identical dimensions (50 mm × 50 mm × 50 mm) were tested according to ASTM D1621-10 [22] to obtain the material properties. The displacement control was adopted. The displacement rate was 0.5 mm per min. The material properties of foam core are summarized in Table 3.

### 2.3. Test Set-Up for Low Velocity Impact

**Figure 3:**
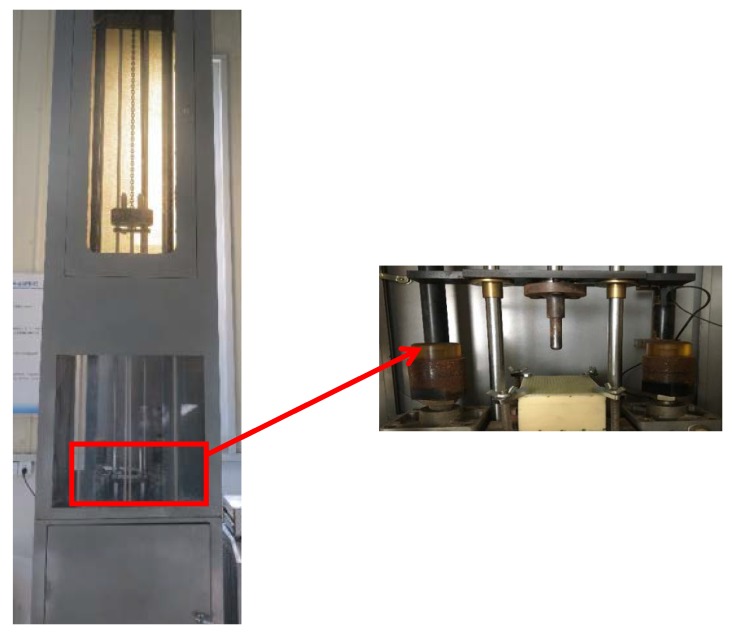
The drop tower apparatus.

The drop tower device with a free-falling mass (4.5 kg) was adopted to impact with the wall boards (see Figure 3). The device can provide a range of 25–2000 J impact energy. Impact loads were measured by a loading transducer. Upon release, the falling mass could fall along two guide rails, and through the center hole of a thick plate to impact on the setting location. Multiple impacts were avoided due to the presence of a rebound brake.

### 2.4. Test Set-Up for Compression after Impact (CAI) and Instrumentation

**Figure 4:**
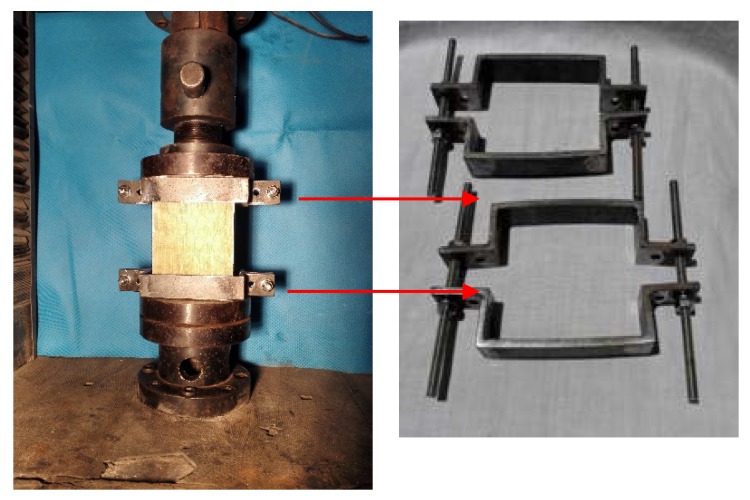
CAI test set-up.

Figure 4 shows the CAI testing set-up. The boundary conditions of specimen are fixed at both ends. A compressive load was applied by a 50 tons capacity hydraulic actuator. The specimen was tested under a displacement control. The displacement rate was 1 mm per min. To measure the axial shortening of the specimen under edgewise compression, two linear variable displacement transducers were employed to record deformation, and another one was placed at the middle height of the specimen to record lateral deformation. For each specimen, the strain gauges were pasted on face sheets to record the longitudinal strain distribution.

## 3. Impact Test Results and Discussion

### 3.1. Effects of Impact Energy

**Figure 5:**
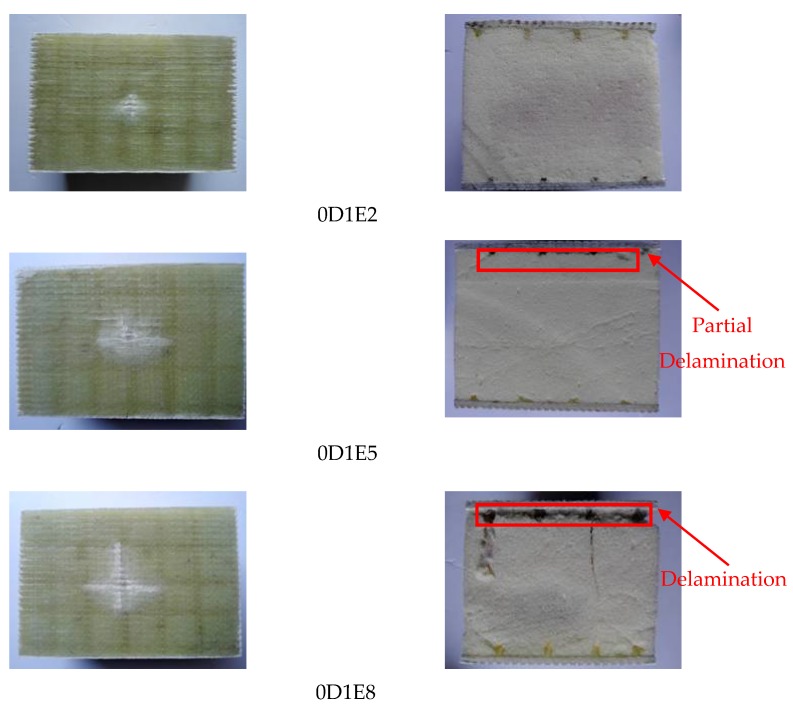
Impact damage with different impact energy.

The side views of specimens 0D1E2 (E = 27 J), 0D1E5 (E = 54 J), and 0D1E8 (E = 81 J), respectively, are shown in Figure 5. For Specimen 0D1E2, the damage region was concentrated with the damage radius of 10 mm, and trace depth was uniform. The GFRP skin was well connected to the foam core; for Specimen 0D1E5, its damage shape was approximate an ellipse. The impact trace was obvious at the impact point, while the peripheral impact traces became blurred. Interfacial fracture cracks were generated. The damage length and width were about 42 mm and 25 mm, respectively; for specimen 0D1E8, a cross-shaped damage region appeared. The length of damage region was over 50 mm. It can be found that the extrusion deformation of foam core occurred, and the shear failure of the foam core appeared. In the meantime, the interfacial delamination occurred. Hence, it can be concluded that impact damage region enlarged with increasing impact energy; moreover, the larger impact energy can lead to the occurrence of interfacial fracture delamination. The impact test results are summarized in Table 4. Compared to Specimen 0D1E2 (v = 2.91 m/s, F = 3.77 kN), the impact velocity (v) and maximum contact fore (F) of Specimen 0D1E5 increased by 44.3% and 41.1%, respectively, which were 4.20 m/s and 5.32 kN; the impact velocity (v) and maximum contact force (F) of Specimen 0D1E8 increased by 78.4% and 72.7%, respectively, which were 5.19 m/s and 6.51 kN. Compared to Specimen L0D1E2 (v = 2.86 m/s, F = 7.84 kN), the impact velocity (v) and maximum contact fore (F) of Specimen L0D1E5 increased by 47.9% and 19.9%, respectively, which were 4.23 m/s and 9.40 kN; the impact velocity (v) and maximum contact force (F) of Specimen L0D1E8 increased by 82.9% and 34.4%, respectively, which were 5.23 m/s and 10.54 kN. Compared to Specimen L1D1E2 (v = 2.89 m/s, F = 11.35 kN), the impact velocity (v) and maximum contact fore (F) of Specimen L1D1E5 increased by 45.7% and 7.0%, respectively, which were 4.21 m/s and 12.15 kN; the impact velocity (v) and maximum contact force (F) of Specimen L1D1E8 increased by 81.0% and 26.9%, respectively, which were 5.23 m/s and 14.40 kN. Compared to Specimen L2D1E2 (v = 2.88 m/s, F = 15.62 kN), the impact velocity (v) and maximum contact fore (F) of Specimen L2D1E5 increased by 45.8% and 13.0%, respectively, which were 4.20 m/s and 17.65 kN; the impact velocity (v) and maximum contact force (F) of Specimen L2D1E8 increased by 80.6% and 33.4%, respectively, which were 5.20 m/s and 20.83 kN. Hence, the larger impact energy can generate a larger impact velocity, and a larger contact force can also be achieved. When the impact energy was double, the impact velocity and larger contact force increased by roughly 40% and 10%, respectively; when impact energy was three times larger, the impact velocity and larger contact force increased by roughly 80% and 30%, respectively.

### 3.2. Effects of Foam Density

**Figure 6:**
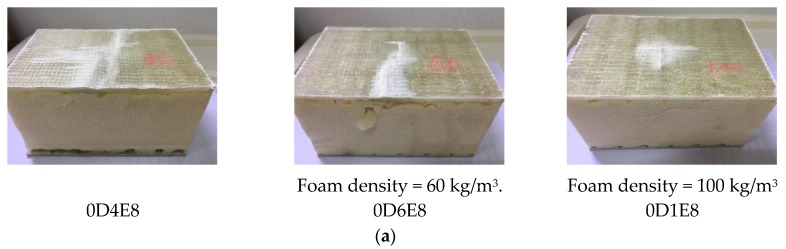

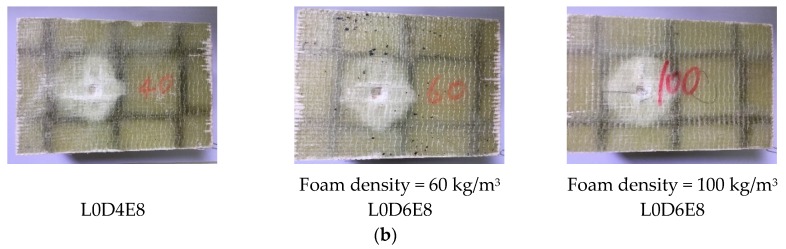
Impact damage with different foam densities.

Figure 6a shows the influences of foam density on impact damage and failure behaviors of specimens without webs. For Specimen 0D4E8, damage region was larger than that of Specimens 0D6E8 and 0D1E8. The upper skin was complete fracture. A large region of interfacial delamination can be found. For Specimen 0D6E8, the upper skin was partial fracture. Interfacial delamination also occurred. For Specimen 0D1E8, a cross-shaped damage region can be found, while interfacial delamination did not occur. Figure 6b shows the influence of foam density on impact damage and failure behaviors of specimens with impact location of L0. For Specimens L0D4E8, L0D6E8, and L0D1E8, the whole damage regions were almost identical, because the two-direction webs restrict the expansion of fiber fracture of upper skin, but the impact region and depth at the impact point decreased with the increase in foam density. Thus, foam density has great effect on resistance to impact damage. The greater foam density, the larger resistance to impact damage.

Table 4 shows the test results of specimens with different foam densities. Compared to Specimen 0D4E8 (F = 3.23 kN), the value of F of Specimen 0D6E8 increased by 18.9%, which was equal to 3.84 kN, and the value of F of Specimen 0D1E8 increased by 101.5%, which was equal to 6.51 kN. Compared to Specimen L0D4E8 (F = 5.73 kN), the value of F of Specimen L0D6E8 increased by 37.2%, which was equal to 7.86 kN, and the value of F of Specimen L0D1E8 increased by 83.9%, which was equal to 10.54 kN. Hence, the larger foam density can alleviate the impact damage, since the foam core can provide much more supporting resistance to the face sheets. Meanwhile, increasing the foam density can lead to a greater contact force. When the foam density was 1.5 times larger, the contact force at least increased by 19%; when impact energy was 2.5 times larger, the contact force at least increased by 84%.

### 3.3. Effects of Impact Location

**Figure 7:**
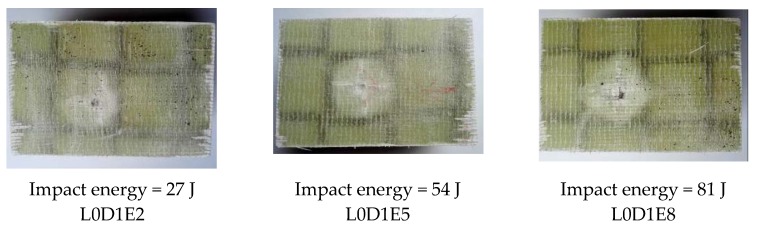
Impact damage at Location L0.

**Figure 8:**
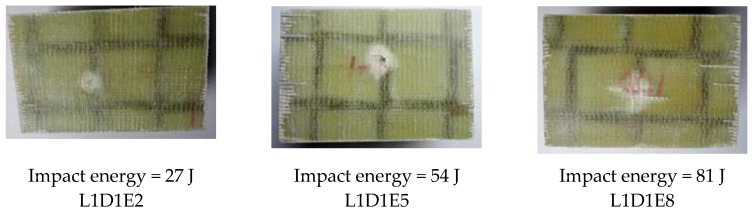
Impact damage at Location L1.

**Figure 9:**
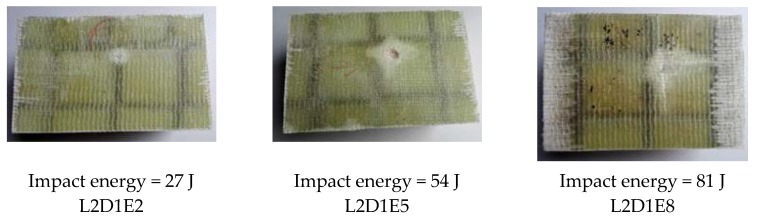
Impact damage at Location L2.

Figure 7 shows the impact damage of specimens with impact location of L0. For all the wall boards, the impact damage region was a square region that was surrounded by the webs. The damage depth of Specimen L0D1E2 was smallest due to the minimum impact energy. For specimen L0D1E5, a cross-shaped impact damage can be found in the damage region. For specimen L0D1E8, the fiber breakage and the penetration of upper skin occurred due to the maximum impact energy. Figure 8 shows the impact damage of specimens with impact location of L1. The damage region and failure behavior of specimens in this group were relatively concentrated. The damage region was small, mainly in the form of dent and crack. With increasing impact energy, cross-shaped impact damage was generated. Figure 9 shows the impact damage of specimens with impact location of L2. For Specimen L2D1E2, the impact surface was flat, and there were almost no dents traces; for Specimen L2D1E5, the impact dent can be found. The damage region had a tendency to spread along the longitudinal and transverse webs; for specimen L2D1E8, the cross-shaped impact damage was generated; and in the meantime, the skin fracture occurred along the directions of the webs. In addition, for all web-foam core specimens, owing to the use of webs, the interfacial delamination did not occur.

Compared to Specimen L0D1E2, the impact force was 7.84 kN. When impact location become L1 and L2, the corresponding impact forces were 11.35 kN for Specimen L1D1E2 and 15.62 kN for Specimen L2D1E2, which increased by 44.8% and 99.2%, respectively. Compared to Specimen L0D1E5, the impact force was 9.40 kN. When impact location become L1 and L2, the corresponding impact forces were 12.15 kN for Specimen L1D1E5 and 17.65 kN for Specimen L2D1E5, which increased by 29.3% and 87.8%, respectively. Compared with L0D1E8, the impact force was 10.54 kN. When impact location become L1 and L2, the corresponding impact forces were 14.40 kN for Specimen L1D1E8 and 20.83 kN for Specimen L2D1E8, which increased by 36.6% and 97.6%, respectively.

**Figure 10:**
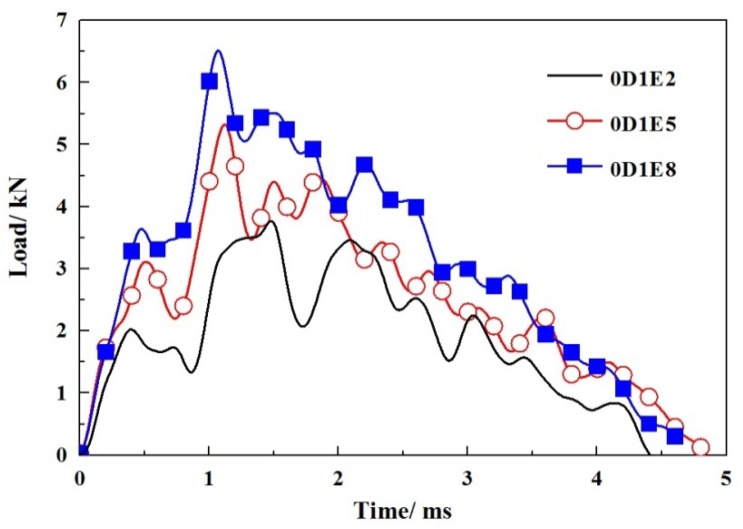
Impact load-time histories of specimens without webs.

**Figure 11:**
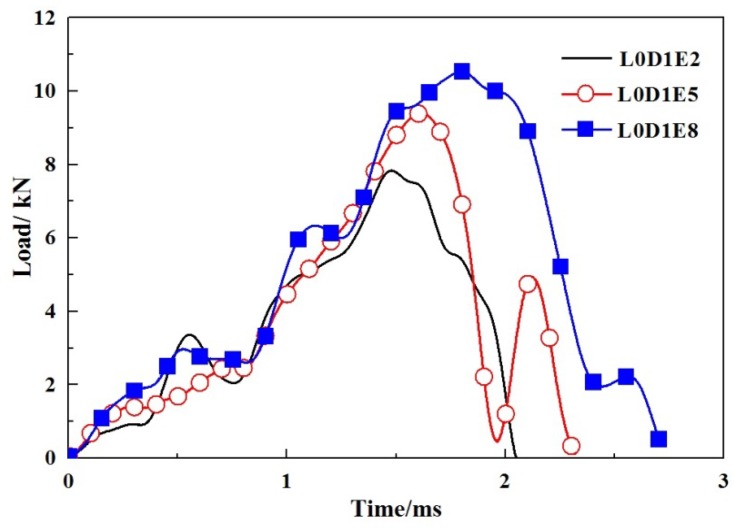
Impact load-time histories of specimens with impact location L0.

**Figure 12:**
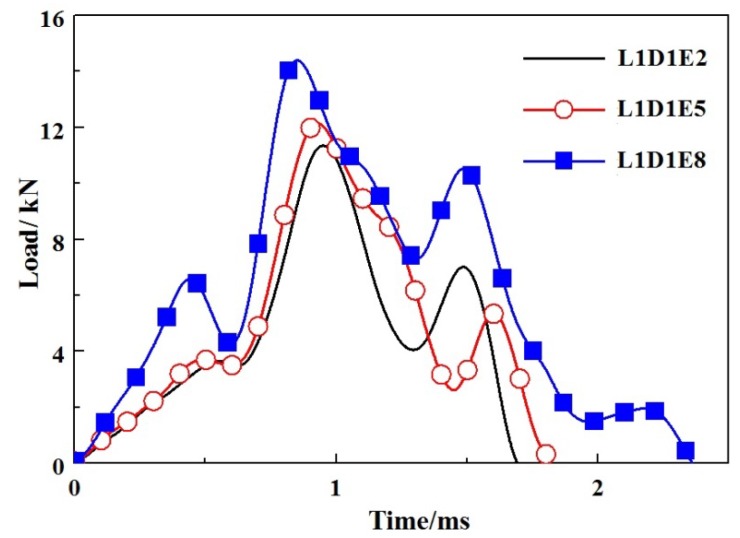
Impact load-time histories of specimens with impact location L1.

**Figure 13:**
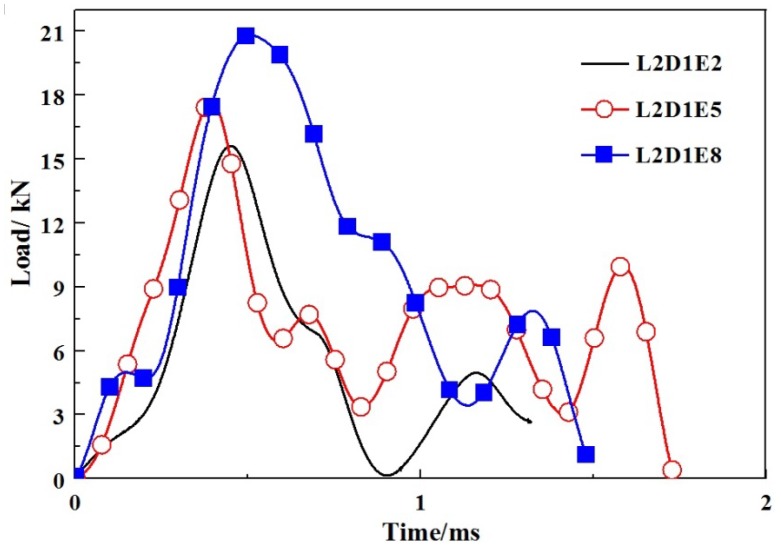
Impact load-time histories of specimens with impact location L2.

According to the impact time history curves, as shown in Figure 10, the impact time of specimens without GFRP webs were usually completed within 5 ms. When an impact event occurred, the impact force reached its maximum value in a short period of time, then fluctuated with decreases step by step. The larger the impact energy, the less the subsequent fluctuations. When the impact location was at L0, as shown in Figure 11, the impact force decreased sharply once it reached the peak value. When the impact location was at L1, the impact forces reached the peak values in a short period, as shown in Figure 12. A fluctuation of impact force emerged in the rapid rise and decline stages, respectively, but the time was short-lived. When the impact location was at L2, the impact forces usually reached the peak values in 0.5 ms, while the decline stages were slow, as shown in Figure 13. It can be concluded that from the perspective of the duration of the impact tests, the duration of specimens impacted on location L2 were fastest, while the duration of specimens without GFRP webs were longest. The reason was that the support of impact location L2 was stiffer than those of impact locations L0 and L1.

**Figure 14:**
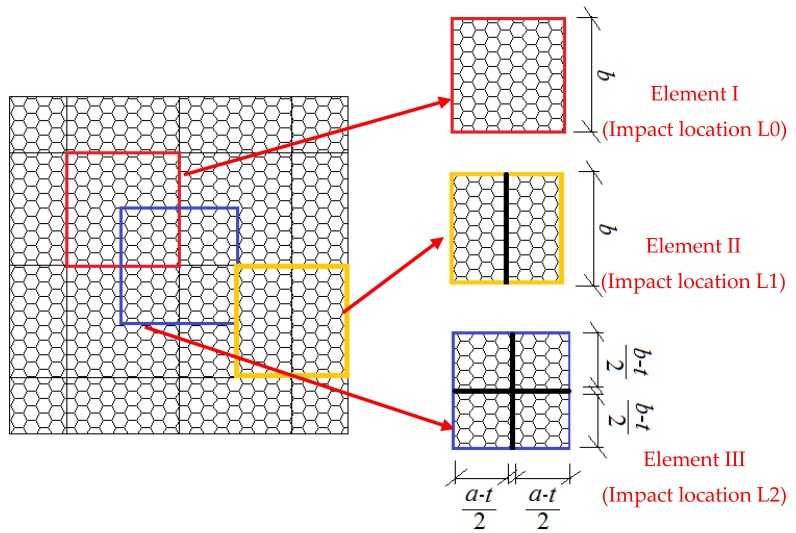
Spring model of core material with different impact locations.

The core material can be represented by a spring with a stiffness of *K* [18]. The web and foam are assumed to have perfect bonding through the resin. Consider a foam-GFRP web core (WFC) element cut out of the panel, as shown in Figure 14; the length and width are *a* and *b*, respectively. The Young’s modulus of core material in the *z*-direction (*E_c_*) can be written as Equation (1) [17].

(1) $$E_{c}=E_{f}\gamma_{f}+E_{w}\gamma_{w}$$

in which *E_f_* and *E_w_* are the Young’s modulus of foam and GFRP web, respectively; *γ_f_* and *γ_w_* are the volume ratio of foam and GFRP web, respectively.

According to the dimension of core elements, the *E_c_* of Element I, II, and III can be expressed as Equations (2)–(4), respectively.

(2) $$E_{c}=E_{f}$$

(3) $$E_{c}=\frac{E_{f}\left(a-t\right)}{a}+\frac{E_{w}t}{a}$$

(4) $$E_{c}=\frac{E_{f}\left(a-t\right)\left(b-t\right)}{ab}+\frac{E_{w}t\left(a+b-t\right)}{ab}$$

Then, the predicted Young’s modulus of core material can be calculated by Equations (2)–(4), which were 15.17 MPa, 309.36 MPa, and 588.11 MPa, respectively. It can be found that the stiffness of Element III was the largest, while the stiffness of Element I was the lowest. Hence, the impact force of specimens with impact location of L2 was larger than those of specimens with impact location of L0 and L1 under the same conditions of impact energy and foam density.

## 4. CAI Testing Program

### 4.1. Intact Wall Board Compression

Two un-damaged specimens (Specimens 0D1E0 and L0D1E0) were tested under axial compressive loading. Two strain gauges were attached on both face sheets of each board to record the axial deformation. The compressive failure of both face sheets occurred together. Two strain gauge readings were very similar until they ultimately failed; hence, the flexible behavior can be ignored. The ultimate compressive loadings of Specimens 0D1E0 and L0D1E0 were 48.62 kN and 103.92 kN, respectively.

### 4.2. Damaged Wall Panel Compression

**Figure 15:**
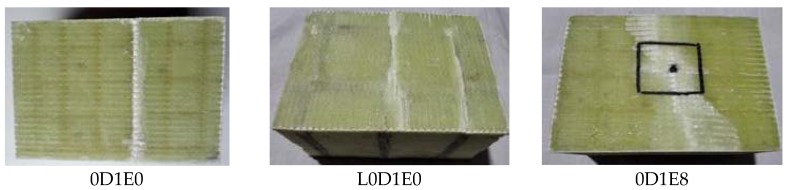

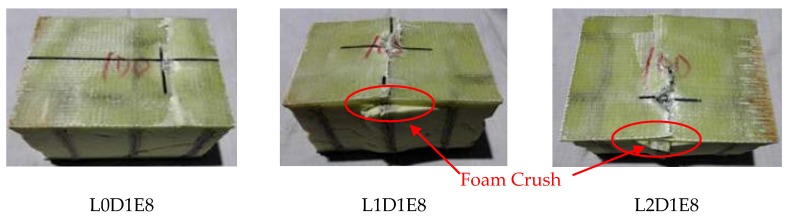
CAI test results.

The test results are shown in the Figure 15. Specimen 0D1E8 appears on the impact point as the center of the crack; both sides of the crack are white, until extended to both sides of the edge. For Specimen L0D1E8, the crack occurred near the edge with crushable tendency. For specimen L1D1E8, the crack occurred at the point of impact, the GFRP face sheet is broken into two parts, and the foam is crushed. Specimen L2D1E8 is similar to specimen L1D1E8, the closer to the impact point, the more serious damage was.

### 4.3. CAI Test Results and Discussion

Four damaged specimens, namely, 0D1E8, L0D1E8, L1D1E8, and L2D1E8, were tested under the axial compressive loading. Compared to the intact specimens, the damaged boards failed only in the impact damaged face sheet. Due to the impact damage, the stiffness of damaged face sheet was lower, which led to the presence of an additional bending moment. Moreover, the strain gauges readings proved that the un-damaged GFRP face sheet continued to apply resistance to the axial compressive loading once the damaged face sheet failed.

**Figure 16:**
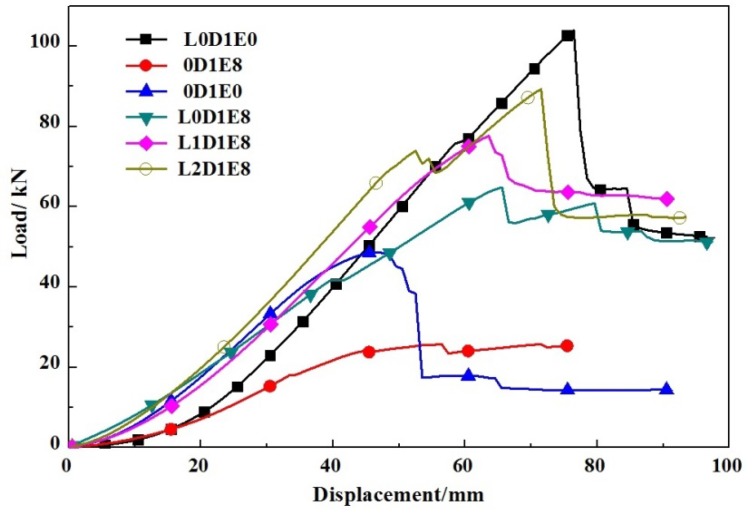
Load-displacement curves at different impact locations.

Table 5 lists the axial strength of all wall boards. As shown in Figure 16, compared to undamaged specimens, all damaged specimens exhibited a sharp decrease in ultimate strength, from 14.1% to 47.0%. For Specimen 0D1E8, the ultimate axial strength was 25.77 kN, compared to 48.62 kN for Specimen 0D1E0, corresponding to a reduction of 47.0%. For Specimens L0D1E8, L1D1E8, and L2D1E8, the ultimate axial strength was 64.83 kN, 77.64 kN, and 89.28 kN, respectively, compared to 103.92 kN for Specimen L0D1E0, corresponding to a reduction of 37.6%, 25.3%, and 14.1%, respectively. Therefore, under the same conditions of impact energy and foam density, the residual axial strength of a specimen with impact location of L2 was largest, while that of a specimen with impact location of L0 was smallest, which was only 28.9% of the residual axial strength of a specimen with impact location of L2. This may be because the area of damage for the region of specimens with impact location of L2 was smallest.

## 5. Conclusions

This study exhibits an experimental investigation on sandwich wall boards with GFRP face sheets and a web-foam core exposed to low-velocity test and compression after impact. The experimental and analytical findings are as follows:

(1) For all specimens, the larger impact energy can generate a larger impact velocity and a larger contact force. For the specimens without webs, the impact damage region enlarged with increasing impact energy, and the larger impact energy can lead to the occurrence of interfacial fracture delamination. For foam-web core specimens, the impact damage region cannot be enlarged due to the restriction of GFRP webs.
(2) The larger foam density can alleviate the impact damage, because the foam core can provide much more supporting resistance to the face sheets. Meanwhile, increasing the foam density can lead to a greater contact force. When the foam density was 1.5 times larger, the contact force at least increased by 19%; when impact energy was 2.5 times larger, the contact force at least increased by 84%.
(3) The impact damage of web-foam core wall boards was affected by the impact locations. For location L0, the damage region usually was a square area surrounded by the webs; for location L1, the failure mode of specimens behaved in the form of dent and crack; and for location L2, the cross-shaped impact damage, as well as the skin fracture along the directions of the webs, can be found.
(4) An analytical model was proposed to predict the Young’s modulus of core material. The values of Young’s modulus of core materials were 15.17 MPa, 309.36 MPa, and 588.11 MPa when the impact locations were L0, L1, and L2, respectively. Hence, it can be concluded that the impact force of specimens with impact location of L2 was larger than those of specimens with impact locations of L0 and L1 under the same conditions of impact energy and foam density. Moreover, the duration of specimens with impact locations of L2 were fastest, while the duration of specimens without GFRP webs were longest.
(5) The residual axial strength of damaged wall boards was evaluated by comparing the control wall boards. With the identical impact energy and foam density, the residual axial strength of a specimen with impact location of L2 was largest, while that of a specimen with impact location of L0 was smallest, which was only 28.9% of the residual axial strength of a specimen with impact location of L2. The reason was that the area of damage region of specimens with impact locations of L2 was smallest.

## Figures and Tables

**Table 1:** Material properties of foams.

Group	Specimen	Foam Density (kg/m^3^)	Impact Location	Impact Energy (J)
A	0D1E0	100	-	0
	0D1E2	100	-	27
	0D1E5	100	-	54
	0D1E8	100	-	81
	0D4E8	40	-	81
	0D6E8	60	-	81
	0D1E8	100	-	81
B	L0D4E8	40	L0	81
	L0D6E8	60	L0	81
	L0D1E0	100	-	0
	L0D1E2	100	L0	27
	L0D1E5	100	L0	54
	L0D1E8	100	L0	81
C	L1D1E2	100	L1	27
	L1D1E5	100	L1	54
	L1D1E8	100	L1	81
D	L2D1E2	100	L2	27
	L2D1E5	100	L2	54
	L2D1E8	100	L2	81

**Table 2:** Material properties of GFRP face sheets.

	Face Sheet	Web
Compressive strength (MPa)	162.8	165.7
Compressive modulus (GPa)	6.26	6.13
Compressive modulus (GPa)	301.5	322.3
(GPa)Tensile modulus (GPa)	6.61	6.57

**Table 3:** Material properties of foams.

Foam Density (*ρ*) (kg/m^3^)	Yield Strength (*f_y_*) (MPa)	Young’s Modulus (*E_f_*) (MPa)
40	0.171	5.02
60	0.366	9.87
100	0.637	15.17

**Table 4:** Impact test results.

Group	Specimen	Velocity (m/s)	Max. Contact Force (kN)
A	0D1E0	-	-
	0D1E2	2.91	3.77
	0D1E5	4.20	5.32
	0D4E8	5.24	3.23
	0D6E8	5.21	3.84
	0D1E8	5.19	6.51
B	L0D4E8	5.22	5.73
	L0D6E8	5.22	7.86
	L0D1E0	-	-
	L0D1E2	2.86	7.84
	L0D1E5	4.23	9.40
	L0D1E8	5.23	10.54
C	L1D1E2	2.89	11.35
	L1D1E5	4.21	12.15
	L1D1E8	5.23	14.40
D	L2D1E2	2.88	15.62
	L2D1E5	4.20	17.65
	L2D1E8	5.20	20.83

**Table 5:** CAI test results.

Specimen Number	Impact Energy (J)	Impact Location	Maximum Contact Force (kN)
0D1E0	0	None	48.62
L0D1E0	0	None	103.92
0D1E8	81	-	25.77
L0D1E8	81	L0	64.83
L1D1E8	81	L1	77.64
L2D1E8	81	L2	89.28
